# Seasonal Dynamics of Soil Labile Organic Carbon and Enzyme Activities in Relation to Vegetation Types in Hangzhou Bay Tidal Flat Wetland

**DOI:** 10.1371/journal.pone.0142677

**Published:** 2015-11-11

**Authors:** Xuexin Shao, Wenying Yang, Ming Wu

**Affiliations:** Wetland Ecosystem Research Station of Hangzhou Bay, Research Institute of Subtropical Forestry, Chinese Academy of Forestry, Fuyang, Zhejiang, China; Tennessee State University, UNITED STATES

## Abstract

Soil labile organic carbon and soil enzymes play important roles in the carbon cycle of coastal wetlands that have high organic carbon accumulation rates. Soils under three vegetations (*Phragmites australis*, *Spartina alterniflora*, and *Scirpusm mariqueter*) as well as bare mudflat in Hangzhou Bay wetland of China were collected seasonally. Seasonal dynamics and correlations of soil labile organic carbon fractions and soil enzyme activities were analyzed. The results showed that there were significant differences among vegetation types in the contents of soil organic carbon (SOC) and dissolved organic carbon (DOC), excepting for that of microbial biomass carbon (MBC). The *P*. *australis* soil was with the highest content of both SOC (7.86 g kg^-1^) and DOC (306 mg kg^-1^), while the *S*. *mariqueter* soil was with the lowest content of SOC (6.83 g kg^-1^), and the bare mudflat was with the lowest content of DOC (270 mg kg^-1^). Soil enzyme activities were significantly different among vegetation types except for urease. The *P*. *australis* had the highest annual average activity of alkaline phosphomonoesterase (21.4 mg kg^-1^ h^-1^), and the *S*. *alterniflora* had the highest annual average activities of β-glycosidase (4.10 mg kg^-1^ h^-1^) and invertase (9.81mg g^-1^ 24h^-1^); however, the bare mudflat had the lowest activities of alkaline phosphomonoesterase (16.2 mg kg^-1^ h^-1^), β-glycosidase (2.87 mg kg^-1^ h^-1^), and invertase (8.02 mg g^-1^ 24h^-1^). Analysis also showed that the soil labile organic carbon fractions and soil enzyme activities had distinct seasonal dynamics. In addition, the soil MBC content was significantly correlated with the activities of urease and β-glucosidase. The DOC content was significantly correlated with the activities of urease, alkaline phosphomonoesterase, and invertase. The results indicated that vegetation type is an important factor influencing the spatial-temporal variation of soil enzyme activities and labile organic carbon in coastal wetlands.

## Introduction

Soil enzymes and soil organic carbon play important roles in the material cycle of wetland ecosystems. Soil enzymes are one of the active organic ingredients in soil, which are closely related to the organic matter decomposition, energy transfer and nutrient cycling[1]. They are mediators and catalysts of biochemical processes that are important in soil functions[2, 3]. Thus, enzyme activities have great potential in providing a unique integrative biological assessment of soils[4]. Soil labile organic carbon refers to the part of organic carbon that is strongly affected by plants or microorganisms, has certain solubility, fast migration rates and poor stability, can be easily oxidized and mineralized in the soil[5]. Soil labile organic carbon not only is a driving force for soil nutrients and the energy in soil microbial activities but also directly gets involved in the soil biochemical conversion process[5, 6]. And it can reflect the minor changes of the soil caused by soil management practices and the environment, prior to the soil total organic carbon (SOC) changes. So soil labile organic carbon can be used as a sensitive index of soil quality and potential productivity[7, 8]. As parts of the soil labile organic carbon, the microbial biomass carbon (MBC) and dissolved organic carbon (DOC) have greater contributions in solving the greenhouse gas emission problem. These two kinds of carbon have more sensitive responses to climate changes[9]. The MBC is the main expression of soil microbial biomass which is the key to control the carbon and other nutrient flows in the ecological system[10], while the formation, migration and transformation of DOC in the soil have important effects on wetland carbon flux. Therefore, shorter term changes in soil quality are often assessed using labile soil organic carbon and enzyme activities.

Wetland is an important carbon pool of the terrestrial ecosystem[11]. The change of soil enzyme activity is bound to change of material circulation process, resulting in the change of the organic carbon especially the labile organic carbon pools in wetland; therefore a lot of researches were carried out to study the influence of soil enzyme activity on soil organic carbon[12–14]. Both the soil enzyme activity and organic carbon could be affected by the changes in vegetation type and environmental factors (such as temperature, moisture, etc.)[15–17], but the researches about the relationship of the two in different seasons and under different vegetation types are relatively rare. As a result, it is very important to study the seasonal variation and relationship between labile organic carbon fractions and enzyme activities such as phosphomonoesterase, urease, β-glycosidase, and invertase which are closely related to the carbon, nitrogen and phosphorus cycles in the wetland ecosystem.

Hangzhou Bay wetland, located in the demarcation line of north and south coastal wetlands of China, is part of the main distribution areas of coastal wetlands. There are only a few plant species in the wetland. Native dominant plant species include *Phragmites australis* and *Scirpus mariqueter*, each of which can form a dense monoculture. In addition, due to the invasion of non-native *Spartina alterniflora*, its mosaic community appeared between *S*. *mariqueter* and *P*. *australis* regions. The area's strong tidal currents have a high carrying capacity for suspended sediment that were carried downstream from the Yangtze River and Qiantang River, and allow rapid accumulation of sediments[18]. The extension of the tidal flat is accompanied with the succession of vegetation. The purpose of this experiment was to determine whether the soil labile organic carbon and enzyme activities were influenced by the variation of vegetation types and seasons. Also this paper discussed the relationship between soil labile organic carbon and enzyme activities in a coastal environment to provide a theoretical basis for further understanding of the nutrient cycling process in Hangzhou Bay coastal wetland.

## Materials and Methods

### Ethics statement

We carried out the study at Wetland Ecosystem Research Station of Hangzhou Bay, which belongs to the Research Institute of Subtropical Forestry, Chinese Academy of Forestry. All necessary permits were obtained for the described field study. This work is unrelated to any ethics issues, and we also confirmed that the field studies did not involve endangered or protected species.

### Site description

**Fig 1:**
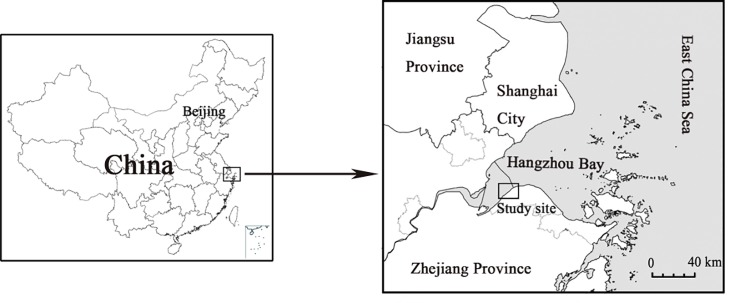
The geographical location of the study area.

The study area is located in the south of Hangzhou Bay (121°09'17″E, 30°19'48″N) and adjacent to the outer part of Qiantang River Estuary (Fig 1). Monocultures of *P*. *australis* are often found around the high marsh zone, while *S*. *mariqueter* dominates the low marsh zone. *S*. *alterniflora* often invades the mid-marsh zone and then quickly expands toward the high and low marsh zones. *P*. *australis* and *S*. *mariqueter* burgeon only in spring, whereas *S*. *alterniflora* has a slow continuous growth in autumn and winter. The biomass of three species showed similar seasonal patterns with a unimodal curve. *P*. *australis* and *S*. *mariqueter* reached the highest biomass in July, whereas *S*. *alterniflora* reached the highest biomass slightly later than *P*. *australis* and *S*. *mariqueter*. The study area is in the southern edge of the northern subtropical region, enjoying a monsoon climate and four distinct seasons, with a mean annual average temperature of 16°C and a mean annual precipitation of 1273 mm. Tidal flat wetlands are greatly influenced by the tides. It is the irregular semidiurnal tide with a reversing current in the area. The rising tide lasts for 6h, the ebb tide lasts for 6.4h, with direction of flood and ebb tidal current is almost parallel to the coastline. Sediments in the study area are mainly silty sand (particle size 3.9–62.5μm). Other basic physiochemical properties of the soil are shown in Table 1.

**Table 1:** Basic physiochemical properties of the soil in the study area.

Types	pH	EC (μs cm^-1^)	Bulk density (g cm^-3^)	TN (g kg^-1^)	TP (g kg^-1^)
***P*. *australis***	8.68±0.49	1.38±0.39	1.07±0.01	0.85±0.10	0.64±0.02
***S*. *alterniflora***	8.77±0.47	1.28±0.34	1.03±0.01	0.62±0.08	0.63±0.02
***S*. *mariqueter***	8.67±0.42	1.88±0.42	1.14±0.01	0.55±0.05	0.62±0.02
**Bare mudflat**	8.75±0.44	2.13±0.55	1.24±0.02	0.48±0.10	0.59±0.03

### Experiment design

The samples had been collected since January 2010, with regular sampling once per month (no collecting in February). And by the end of December 2010, there were a total of11 times of sample collecting. Three randomly selected 1×1 m plots were selected representative of each vegetations (*P*. *australis*, *S*. *alterniflora*, and *S*. *mariqueter*) as well as bare mudflat separated by at least 100 m apart. The mean vegetation coverage investigated in July was 95.0%, 92.1% and 60.1%, the mean height was 2.11, 1.86 and 0.52 m, the mean aboveground biomass was 3731.7, 2903.9, 487.4g m^-2^, and the mean belowground biomass was 457.2, 459.9, 172.7g m^-2^ for *P*. *australis*, *S*. *alterniflora*, and *S*. *mariqueter*, respectively. Every month before sampling, the un-decomposed litter layers were removed first, and then the multi-point mixed sampling method was employed. The researchers collected the undisturbed soil samples at the depth of 0–20 cm using Eijkelkamp soil sampler and layered them as surface layer of 0–5 cm and root system distribution layer of 5–20 cm. The latter was a mix of rhizosphere and bulk soil but near the plant roots with a distance of about 3 cm. The samples were placed separately into aseptic bags, and then brought back to the laboratory immediately. Plant and animal residues and stone pieces were excluded, proper amount of soil samples were taken using the quartering method and divided into two parts. One part was refrigerated to determine soil moisture content, DOC, MBC and soil enzyme activities, while the other part was air-dried first then ground and sieved to determine pH, electrical conductivity (EC) and SOC.

### Analytical procedures

Alkaline phosphomonoesterase activity was determined by using 4-Nitrophenyl phosphate disodium as a substrate[19, 20]. After incubation at 37°C for 1 h, the amount of *p*-Nitrophenol was measured at 410 nm. Urease activity was measured using urea as a substrate[19, 20]. After incubation at 37°C for 24 h, the amount of NH_4_^+^ was measured at 578 nm. β-glucosidase activity was measured using *p*-nitrophenyl-β-d-glucopyranoside as a substrate[19, 20]. After incubation at 37°C for 1 h, the amount of *p*-nitrophenol (PNP) was measured at 410 nm. Invertase activity was determined by using sucrose as a substrate[19, 20]. After incubation at 37°C for 24 h, the amount of glucose was measured at 508 nm.

Soil DOC was determined following the procedures of Jones and Willett[21]. Moist soil samples (equivalent to 10g oven-dried weight) were extracted with 80 mL of distilled water in a flask by shaking for 30 min at approximately 200 rpm. Next, the samples were centrifuged for 20 min at 3,500 rpm. The supernatant was filtered through 0.45 μm filters into separate vials for C analysis. Soil MBC was measured using the fumigation–extraction method[22]. The fumigated and non-fumigated moist soils were extracted with 0.5 mol L^-1^ K_2_SO_4_ by shaking for 30 min. Carbon content in the extracts was analyzed by TOC-Vcph (Shimadzu, Japan). Soil DOC was calculated by taking the difference between the total dissolved C and the dissolved inorganic C[23]. Soil MBC was calculated using the following equation: MBC = (C_fumigated_-C_non-fumigated_)/0.38[14]. Soil organic carbon content was measured by a K_2_CrO_7_-H_2_SO_4_ oxidation procedure[24].

Soil water content (SWC) was measured by oven drying method. Soil pH was measured in 1:2.5 soil:water suspension and EC in 1:5 soil:water suspension using the PHS-3C pH Meter and DDSJ-308A Conductivity Meter, respectively (Shanghai Precision & Scientific Instrument Co., Ltd. of China)[24].

### Data analysis

The soil labile carbon distributions under various vegetation types were plotted using SigmaPlot 12.0 software. The statistical analysis carried out using SPSS16.0 (StatSoft Inc. 2007). And the Two-way ANOVA analysis method (LSD multiple comparison) was employed to analyze different sampling times and vegetation types. The dependent variables were contents of SOC, MBC, and DOC; and activities of alkaline phosphomonoesterase, urease, β-glycosidase, and invertase. The data was log-transformed when necessary, to achieve homogeneity of variance (Levene's test). When data did not fulfill the sphericity requirement for Maulchly's sphericity test, univariate F statistics using a corrector index of epsilon were applied, based on either Greenhuse–Geisser, Huynh–Feldt or Lowerbound corrections. Paired-Sample T test was applied for different soil layers. And the Pearson’s correlation analysis was used to analyze the correlation between soil enzyme activities and soil labile organic carbon. Differences were considered significant if probabilities for non-significance were <0.05.

## Results

### Seasonal dynamics of soil labile organic carbon in different vegetation types

#### Soil total organic carbon

**Fig 2:**
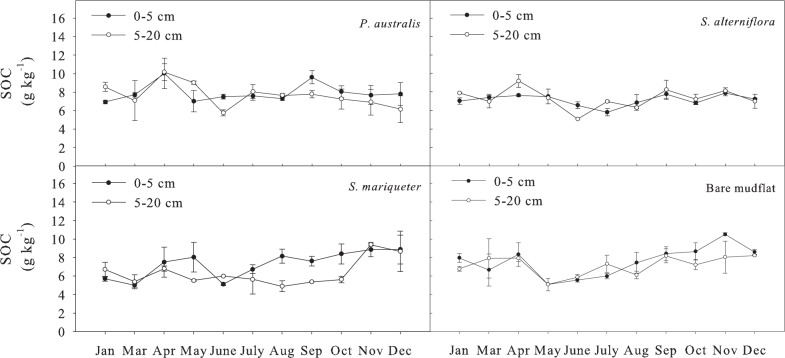
Seasonal dynamics of SOC content in soils under different vegetation types.

The order of annual average SOC levels of different vegetation types in soil with depth of 0-20cm was (Fig 2): *P*. *australis* (7.86 g kg^-1^) >bare mudflat (7.52 g kg^-1^) >*S*. *alterniflora* (7.27 g kg^-1^) >*S*. *mariqueter* (6.83 g kg^-1^). Analysis of variance showed that the difference in SOC content between *P*. *australis* and bare mudflat was not significant (*P*>0.05), but significantly higher than that of *S*. *alterniflora* and *S*. *mariqueter* (*P*<0.05). The seasonal dynamics of SOC content with different vegetation types were significant (*P*<0.01) but with small annual variation coefficient, which was around 16%. The Paired-Sample T test showed that the SOC content between depth of 0–5 cm and 5–20 cm was not significant.

#### Soil microbial biomass carbon

**Fig 3:**
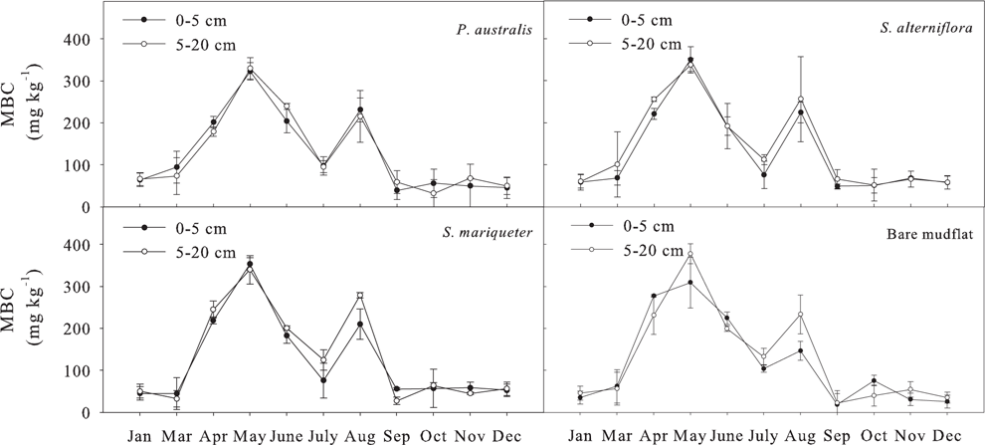
Seasonal dynamics of MBC content in soils under different vegetation types.

The order of annual average MBC of the soils with different vegetation types at the depth of 0–20 cm was (Fig 3): *S*. *alterniflora* (134 mg kg^-1^) >*S*. *mariqueter* (127 mg kg^-1^) >*P*. *australis* (127 mg kg^-1^) >bare mudflat (117 mg kg^-1^). Analysis of variance showed that the differences in MBC content among different vegetation types were not significant, but the seasonal dynamics of MBC content were significant (*P*<0.01) with the annual variation coefficient being close to 80%. The changing curve had two peaks, the maximum value was at the spring (May), and another peak was in the summer (August), while the lowest content was at the winter. The Paired-Sample T test (Table 2) showed that MBC content of 5–20 cm was significantly higher than that of 0–5 cm (*P*<0.05).

**Table 2:** Variations of SOC, MBC and DOC contents in soils under different vegetation types.

	SOC (g kg^-1^)		MBC (mg kg^-1^)		DOC (mg kg^-1^)	
	0–5 cm	5–20 cm	0–5 cm	5–20 cm	0–5 cm	5–20 cm
**Mean**	7.48a	7.16a	125b	133a	284b	308a
**Std.**	1.22	1.27	98.1	104	93.7	113
**C.V.**	16.3%	17.7%	78.7%	77.8%	33.0%	36.6%

Note: Different letters in the same row represent significant differences (*P*<0.05).

#### Soil dissolved organic carbon

**Fig 4:**
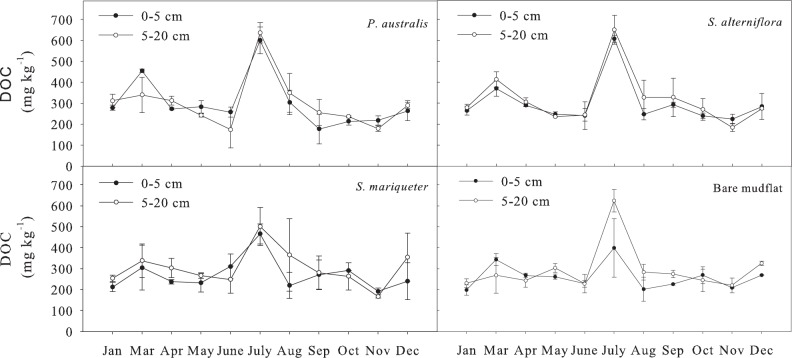
Seasonal dynamics of DOC content in soils under different vegetation types.

The order of annual average DOC of different vegetation types with soil depth of 0–20 cm was (Fig 4): *P*. *australis* (306 mg kg^-1^) >*S*. *alterniflora* (300 mg kg^-1^) >*S*. *mariqueter* (289 mg kg^-1^) > bare mudflat (270 mg kg^-1^). Analysis of variance showed that the difference between bare mudflat and *S*. *mariqueter* soil was not significant, but was significantly lower than that of *P*. *australis* and *S*. *alterniflora* (*P*<0.05). The DOC content changed significantly with season (*P*<0.01). The soil DOC content was the highest in July of summer. And the change remained relatively stable in the other months of the year. The variation coefficient of soil DOC content was around 35%, less than that of soil MBC content. The DOC content of 5–20 cm was significantly higher than that of 0-5cm (*P*<0.05).

### Seasonal dynamics of soil enzyme activities in different vegetation types

#### Soil alkaline phosphomonoesterase activity

**Fig 5:**
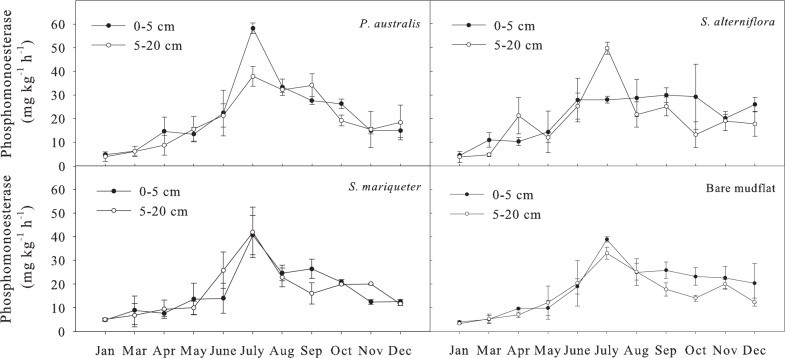
Seasonal dynamics of alkaline phosphomonoesterase activity in soils under different vegetation types.

The order of annual average activity of alkaline phosphomonoesterase for the soil of 0–20 cm depth with different vegetation types was (Fig 5): *P*. *australis* (21.4 mg kg^-1^ h^-1^) >*S*. *alterniflora* (19.1 mg kg^-1^ h^-1^) >*S*.*mariqueter* (17.7 mg kg^-1^ h^-1^) >bare mudflat (16.2 mg kg^-1^ h^-1^). Analysis of variance showed that the difference in alkaline phosphomonoesterase activity between bare mudflat and *S*. *mariqueter* was not significant, but significantly lower than that of *P*. *australis* and *S*. *alterniflora* (*P*<0.05). The seasonal dynamic of alkaline phosphomonoesterase activity was significant (*P*<0.01). The soil enzyme activity was the highest in July of summer, while was the lowest in January of winter. The variation coefficient of soil alkaline phosphomonoesterase activity was >50%. The Paired-Sample T test showed no significant difference between depth of 0–5 cm and 5–20 cm.

#### Soil urease activity

**Fig 6:**
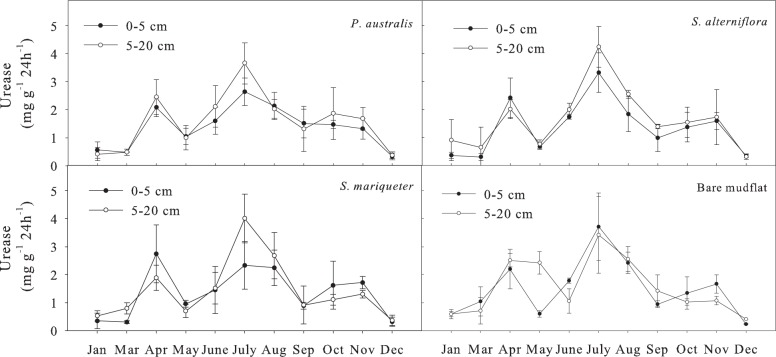
Seasonal dynamics of urease activity in soils under different vegetation types.

The order of annual average urease activity of different vegetation types at the soil depth of 0–20 cm was (Fig 6): *P*. *australis* (1.59 mg g^-1^ 24h^-1^) >*S*. *alterniflora* (1.49 mg g^-1^ 24h^-1^) >*S*.*mariqueter* (1.38 mg g^-1^ 24h^-1^)>bare mudflat (1.34 mg g^-1^ 24h^-1^). Analysis of variance showed that there was no significant difference in urease activity among different vegetation types. But the seasonal variation of urease activity was significant (*P*<0.01). There were two peaks in the changing curve, the maximum value appeared in the summer (July), the other peak appeared in the spring (April), and the lowest content was observed in winter. The annual variation coefficient of soil urease activity was>60% (Table 3) with a relatively big amplitude, slightly higher than that of alkaline phosphomonoesterase. The Paired-Sample T test showed that there was no significant difference between the two layers.

**Table 3:** Annual variation coefficients of enzyme activities in soils.

	Phosphomonoesterase (mg kg^-1^ h^-1^)		Urease (mg g^-1^ 24h^-1^)		β-glycosidase (mg kg^-1^ h^-1^)		Invertase (mg g^-1^ 24h^-1^)	
	0–5 cm	5–20 cm	0–5 cm	5–20 cm	0–5 cm	5–20 cm	0–5 cm	5–20 cm
**Mean**	19.5a	18.6a	1.40a	1.56a	3.17a	3.40a	8.73a	8.91a
**Std.**	11.2	11.8	0.87	1.01	2.93	2.76	5.55	6.37
**C.V.**	57.70%	63.20%	62.00%	64.90%	92.40%	81.20%	63.60%	71.40%

Note: Significant differences were indicated by differentletters after values in eachrow (*P*<0.05).

#### Soil β-glycosidase activity

**Fig 7:**
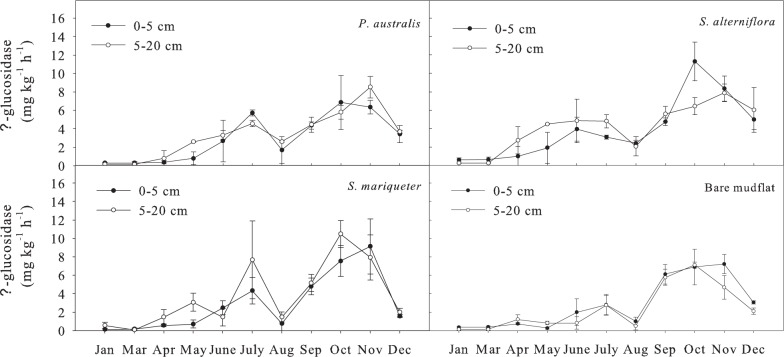
Seasonal dynamics of β—glycosidase activity in soils under different vegetation types.

The order of average β-glycosidase activity of soils with different vegetation types at the depth of 0-20cm was (Fig 7): *S*. *alterniflora* (4.10 mg kg^-1^ h^-1^) >*S*. *mariqueter* (3.59 mg kg^-1^ h^-1^) > *P*. *australis* (3.30 mg kg^-1^ h^-1^) >bare mudflat (2.87 mg kg^-1^ h^-1^). Analysis of variance showed that the β-glycosidase activity of *S*. *alterniflora* soil was significantly higher than that of the others. And the seasonal variation of β-glycosidase activity was significant (*P*<0.01). The soil β-glycosidase activity was lower from January to May, and it began to rise from June, reaching the first peak in July and decreased in August, and then rose sharply, reaching its second peak between October and November. The variation coefficient of β–glycosidase in soil was > 80% (Table 3), being with the largest amplitude among the four enzyme activities. The Paired-Sample T test showed no significant difference between the two layers.

#### Soil invertase activity

**Fig 8:**
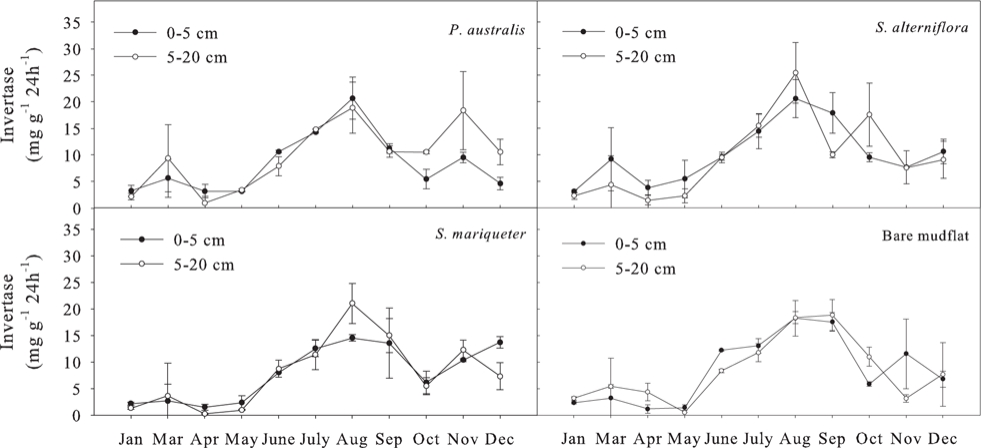
Seasonal dynamics of invertase activity in soils under different vegetation types.

The order of average invertase activity in different wetland vegetation types at soil depth of 0-20cm was (Fig 8): *S*. *alterniflora* (9.81 mg g^-1^ 24h^-1^) >*P*. *australis* (9.80 mg g^-1^ 24h^-1^) >*S*. *mariqueter* (8.22 mg g^-1^ 24h^-1^)>bare mudflat (8.02 mg g^-1^ 24h^-1^). Analysis of variance showed that the invertase activity of *S*. *mariqueter* and bare mudflat were significantly lower than that of *S*. *alterniflora* and *P*. *australis* (*P*<0.05). The seasonal variation of soil invertase activity was significant (*P*<0.01). The change trend was relatively consistent with that of urease, but the invertase activity peak appeared in the summer (August). The annual variation coefficient of soil invertase activity was >60%, close to that of urease, with a relatively large amplitude. The Paired-Sample T test showed no significant difference between the two layers.

### The relationship among soil physiochemical properties, the enzyme activities and the soil organic carbon fractions

A correlation analysis (Table 4) showed that the SOC was significantly positively correlated with the SWC. The DOC was significantly negatively correlated with the SWC and EC, while there was no significant correlation between pH and soil organic carbon fractions. There were no significant correlations between four enzyme activities and EC. The urease had a significantly negative relation with the SWC while the other three enzymes were not. However, the urease and soil pH had no correlation, while the other three enzyme activities and soil pH were significantly positively correlated. Correlations between SOC and DOC, MBC and DOC were not significant, but the SOC and MBC showed a significantly negative correlation. SOC and alkaline phosphomonoesterase, urease, β-glycosidase, and invertase were not significantly correlated, while MBC was significantly positively correlated with urease, and significantly negatively correlated with β-glycosidase. DOC was significantly positively correlated with the enzyme activities except for β-glycosidase.

**Table 4:** The correlation of soil physiochemical properties, enzyme activities, and organic carbon and its labile fractions.

	SWC	pH	EC	Phosphomonoesterase	Urease	β-glucosidase	Invertase	SOC	MBC	DOC
**SWC**	1									
**pH**	-0.139	1								
**EC**	0.083	-0.399**	1							
**Phosphomonoesterase**	-0.075	0.181*	0.027	1						
**Urease**	-0.150*	0.072	0.027	0.523**	1					
**β-glucosidase**	-0.047	0.408**	-0.096	0.400**	0.12	1				
**Invertase**	-0.025	0.284**	0.055	0.544**	0.352**	0.289**	1			
**SOC**	0.256**	0.112	0.054	-0.024	0.002	0.134	-0.026	1		
**MBC**	-0.13	-0.069	0.14	-0.022	0.252**	-0.298**	-0.076	-0.167*	1	
**DOC**	-0.137*	-0.063	-0.165*	0.357**	0.477**	-0.052	0.253**	-0.115	-0.036	1

*P<0.05

** *P*<0.01 (significant at these levels).

## Discussion

### The influence of vegetation types on soil labile organic carbon and enzyme activities

Soil organic carbon content is the result of dynamic balance between the input and output of SOC under the influence of various factors such as the soil, climate, vegetation, and human disturbance[25]. Vegetation mainly affects the input quantity and quality of the SOC. This study showed that the order of SOC content in soil was: *P*. *australis*>*S*. *alterniflora*>*S*. *mariqueter*, which indicated that during the succession from *S*. *mariqueter* to *P*. *australis*, the soil carbon sequestration ability was increasing. MBC and DOC are the main characteristic indexes of soil labile organic carbon[2]. This study showed that the MBC and DOC contents of bare mudflat were lower than that of the soil covered with plants. This may be related to the presence of the plants[16]. The plant's underground root is the energy mechanism of the microbial activity. The change of soil physiochemical property and root exudates will eventually affect the quantity and activity of microorganism[26],and then causes chain variation of the soil components[27]. Sanaullah et al.[15] reported that MBC was significantly higher in vegetated soils than in the unvegetated control, and the difference was probably caused by rhizodeposition. Khalid et al. [28] also reported that the biodegradability of DOC in the soil solution was greater in the unplanted soil than in the planted soil.

Vegetation can also influence soil enzyme activities[16, 20, 29]. Except for urease, the enzyme activities were significantly different among different vegetation types in the study area. Generally, the enzyme activities of the soils under *P*. *australis* and *S*. *alterniflora* were higher than that of *S*. *mariqueter* and bare mudflat. This may be related to the fact that the former two have larger biomass and more developed aerenchyma in both aboveground and belowground parts. The vegetation controls above- and below-ground litter quantity and quality[29], and can excrete exogenous enzymes and affect microbial species composition and diversity by releasing exudates and oxygen into the rhizosphere, which in turn indirectly affect enzyme activities[30, 31]. Therefore, phosphomonoesterase, invertase, and β-glucosidase were closely related to plant growth and the biomass amount. Niemi et al.[32] researched 12 soil enzymes and plant root growth, and also found a positive correlation between plant root biomass and enzyme activities. The soil urease activity and MBC content were significantly positive correlated. As soil enzymes mainly derived from soil microorganisms and other organic tissues (such as plant)[19, 20], the result indicating that the plants growth had a smaller influence on urease, while the microbial activities were probably the main source of urease production to the soils. There were also noticeable seasonal changing patterns of soil enzyme activities. They were generally at the lowest level in the winter (January) and then increased gradually in the study area with the highest activity point appeared in the summer or autumn. Therefore, seasonal dynamics of soil enzyme activities were similar to that of the aboveground plants which sprout in spring (March to May), reached the maximum biomass in summer and had increasing amounts of litters in fall[33].

### The factors affecting correlation between soil labile organic carbon and enzyme activities

Soil enzymes are involved in all the biochemical processes of soil environment. They are the core mediators of soil biochemical process, and are closely related to the organic matter decomposition, energy transfer, nutrient cycling, and environmental quality [1]. In this study, MBC had an extremely significantly negative correlation with the β-glycosidase activity and had no significant correlation with phosphomonoesterase and invertase activities. A suite of soil enzymes operate cooperatively for the organic matter decomposition. Each enzyme has its own substrate and ability to catalyze specific biochemical reactions[23]. The differences in the sources of substrate availability and composition may lead to the changed behaviors of the activity of hydrolytic enzymes, such as phosphomonoesterase, urease, and β-glucosidase in soils. The enzymatic disparity of enzyme activities was also revealed in other study[23, 34, 35]. In this study, DOC content had a significantly positive correlation with the seasonal dynamics of alkaline phosphomonoesterase, urease, and invertase activities in the soil, which means that the production of soil DOC in Hangzhou Bay wetland was positively affected by these enzyme activities. This is due to that extracellular hydrolytic and oxidative enzyme activities are in charge of the conversion of organic matter from high to low molecular weight compounds found in DOC [35, 36]. In addition, DOC contains substrates for enzymatic reactions of varying molecular weight[37]. Fenner et al. [38] and Song et al. [23] also found positive correlations between soil enzyme activities and DOC pools proposing that these enzymes were crucial in mobilizing DOC in peat wetland. However, our results were the opposite to that of research conducted in the Sanjiang Mire Wetland[39]. This may be because our study carried out in a coastal wetland that was under the alternate influence of wetting and drying, leaving the DOC content increased and the enzyme activities improved in the soil. There was no significant correlation between DOC and β-glucosidase in the study area, as β-glucosidase catalyzes the production of glucose from oligosaccharides, while the product could be rapidly consumed through microbial assimilation and metabolism, leading to a decline in DOC[35]. Laboratory studies had also demonstrated that both positive and negative relationships exist between soil enzyme activities and DOC[37]. In short, the soil enzymes' involvement in biogeochemical cycles of the soil will result in the transforming and cycling of soil labile organic carbon pool.

Tidal cycle is one of the main hydrologic processes of coastal wetlands. The SOC content of bare mudflat was significantly higher than that of the *S*. *mariqueter* and *P*. *australis* in this study. The reasons may be that: firstly, the litters run off the water by tide washing with only a small amount remained in the surface soil under *P*. *australis* or *S*. *mariqueter*, while some litters can be left on the bare mudflat and finally returned to the soil. Secondly, the ocean source may also lead to the higher SOC content in the bare mudflat that located in the low zone [40]. The SOC is composed of diverse fractions varying in their degree of decomposition, recalcitrance, and the turnover rate, and the labile organic carbon fractions have been suggested as an early and sensitive indicator of SOC changes[41, 42]. However, there was no significant correlation between DOC and SOC in our results. This may be caused by the tides in the study area. As DOC mainly comes from the recent plant litters and soil organism humus, which are not stable and easy to be leached away[43]. Soil MBC and SOC were with significantly negative correlation. Soil microorganism is the decomposer of soil organic matters. The reduction of microorganism activity leads to the decrease of MBC content and SOC decomposition rates, thus increases the accumulation of SOC. Keuskamp et al. [44] have demonstrated that the microbial activity was primarily inhibited by limited energy, despite a large amount of organic carbon in the mangrove sediment. Nie et al. [45] have reported the different relationship between MBC and SOC in gentle and steep slope landscapes.

Salinity has been proven to negatively influence the size and activity of soil microbial biomass and biochemical processes essential for maintenance of soil organic matter[46, 47]. In our study, soil EC was significantly negatively correlated with DOC, due to solubilization of organic matter and a decrease of microbial activity in sodic and saline soils[48]. While there was no detrimental impact of soil salinity on the enzyme activities compared to the other studies[47, 49]. Soil water content and pH value in the study area had significant correlation with soil enzyme activities (Table 4), suggesting that temperature, moisture, pH, nutrient status, and vegetation cover were the control factors of soil enzyme activities[23, 50, 51]. These seasonal dynamics of the soil enzyme activities, therefore, were a comprehensive response to environmental factors.

## Conclusions

This study revealed that the vegetation types of Hangzhou Bay tidal flat wetland had significant influences on the SOC and DOC and an insignificant influence on the MBC. The annual variation of MBC was the largest, followed by DOC and then SOC, which indicated that labile organic carbon fractions were more sensitive to environmental changes than SOC. Among the enzymes studied urease was mainly affected by microbial activities, while phosphomonoesterase, β-glycosidase, and invertase were closely correlated with plant growth. There were also significant seasonal dynamics among different enzyme activities. In conclusion, variations of vegetation types and seasons had significant impacts on soil labile organic carbon fractions and enzyme activities, and seasonal variation of plant biomass was the potential driving factor for them that were associated with C, N and P cycling in wetlands.

## Supporting Information

S1 Table Soil labile organic carbon contents and soil enzyme activities under different vegetation types and seasons (XLS)
